# Chemical Composition and Cytotoxic Activity of Extracts from *Carpesium divaricatum*: In Vitro- versus Field-Grown Plants

**DOI:** 10.3390/plants11212815

**Published:** 2022-10-23

**Authors:** Janusz Malarz, Agnieszka Galanty, Anna Stojakowska

**Affiliations:** 1 Maj Institute of Pharmacology, Polish Academy of Sciences, Smętna Street 12, 31-343 Kraków, Poland; 2 Department of Pharmacognosy, Jagiellonian University Medical College, Medyczna Street 9, 30-688 Kraków, Poland

**Keywords:** *Carpesium*, cytotoxicity, micropropagation, multiple shoots, plant tissue culture, sesquiterpene lactones, thymol derivatives

## Abstract

*Carpesium* *divaricatum* Sieb. & Zucc. is a plant species rich in terpenoids of anti-inflammatory and cytotoxic activity, especially germacranolides of potential medicinal value. The present study describes in vitro multiplication of *C. divaricatum*, analysis of active constituents in the multiple shoots, and assessment of cytotoxic activities of extracts prepared from in vitro- and field-grown plants. The plant extracts were evaluated for cytotoxicity using two melanoma cell lines (HTB140 and A375); human keratinocytes (HaCaT); two colon cancer cell lines (Caco2 and HT29); human hepatocellular carcinoma cells (HepG2); two lines of prostate cancer cells (DU145 and PC3) and prostate epithelial cells (PNT2). Chemical compositions of the assayed extracts were analyzed by HPLC/DAD, in reference to isolated compounds. Maximum of 4.07 ± 1.61 shoots regenerated from a nodal explant of *C. divaricatum*, cultivated in a liquid MS medium supplemented with thidiazuron (1 μM). In vitro grown shoots and plantlets of *C. divaricatum* accumulated terpenoids that are known as active constituents of the intact plant. Cytotoxic activity of the extracts prepared from the in vitro cultured plants was like that demonstrated by the extracts prepared from field-grown plants and seemed to be more selective than cytotoxicities of the individual germacranolides.

## 1. Introduction

Except for the widespread *C. cernuum* L. and *C. abrotanoides* L., the genus *Carpesium* L. (Asteraceae, Inuleae-Inulinae) comprises ca. 20 Asian species, including six endemics in China [1,2]. Plants of *Carpesium* spp. are rich in sesquiterpene lactones and monoterpenoid thymol derivatives of well documented anti-inflammatory and cytotoxic activity [3,4]. Germacranolides isolated from the plants were frequently studied in respect of their cytotoxic activity against human cancer cells in vitro [5,6,7,8], but only a few studies dealt with the molecular mechanism of action of the compounds [9,10,11]. Recently, a paper reporting on poor selectivity of the cytotoxic effect exerted by *Carpesium* germacranolides has been published [12]. This may raise a question about the safety of internal use of the plant and its preparations. Despite a long-lasting traditional use of *C. divaricatum* Sieb. & Zucc. plants as both medicines and food, correlations between the chemical composition of the plant extracts and their biological activity are poorly investigated.

Though plant tissue culture offers a good experimental system for studying biosynthesis of biologically active plant metabolites and mechanisms of its regulation, there are no data on the production of biologically active terpenoids by in vitro cultures of *Carpesium*. The only study on in vitro culture of *Carpesium* spp. [13] described an effect of light quality on growth and selected physiological parameters (photosynthetic pigments, antioxidant enzymes, radical scavenging activity) of *C. triste* Maxim. plants cultivated in vitro and did not contain any data on biologically active mono- and sesquiterpenoids. Multiple shoots of *Telekia speciosa* (Schreb.) Baumg. (Asteraceae, Inuleae-Inulinae), a species closely taxonomically related to *C. divaricatum* [14,15] produced both thymol derivatives and sesquiterpene lactones characteristic of the parent plant [16,17].

The present study was aimed at assessing productivity of in vitro cultured shoots and/or plantlets of *C. divaricatum* with particular interest in sesquiterpene lactones and thymol derivatives of known biological activity. Cytotoxic activities of extracts from in vitro cultured plant material and from the leaves and roots of the field-grown plants were investigated to compare the effects exerted by the extracts on different cancer and normal cell lines.

## 2. Results

### 2.1. In Vitro Culture

Shoot culture of *C. divaricatum* was initiated from nodal explants excised from aseptic seedlings. Two liquid and three solidified nutrient media of different composition were used for in vitro proliferation of shoots. Two illumination regimes were also applied (see Table 1). After 8 weeks of culture, the number of shoots regenerated from each nodal explant and dry weight (DW) of shoots/plantlets developed from each explant was assessed. Up to the 7th week of culture, the multiple shoots obtained from nodal explants did not develop roots. At the end of the 8-week culture period, spontaneous rooting occurred at a low frequency. The maximum number of shoots (4.07 ± 1.61), as well as the maximum accumulation of the biomass (ca. 0.27 g DW), were achieved in liquid MS medium [18], supplemented with 1 µM TDZ (thidiazuron). The culture was aerated by shaking (100 r.p.m.) and maintained at 28 ± 3 °C, in a photoperiod of 16/8 (light/dark). Comparable results were obtained with the liquid medium containing a diminished concentration of TDZ.

Cultures grown on the media solidified with agar grew slower (up to 0.13 g DW after 8 weeks). Moreover, though significant differences in the number of shoots regenerated from one explant were not observed, the cultures on solidified media tended to produce fewer shoots. There was no visible difference in the leaf color of shoots grown in different illumination regimes.

### 2.2. Phytochemical Analysis

**Figure 1:**
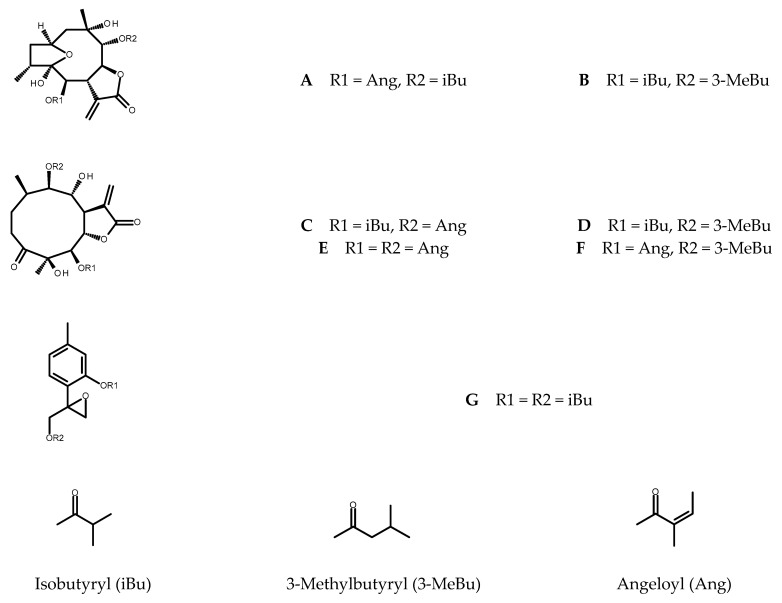
Chemical structures of the terpenoids identified in *C. divaricatum* extracts: isocardivarolide C (**A**); 2*α*,5-epoxy-5,10-dihydroxy-6*α*-isobutyryloxy-9*β*-(3-methylbutyryloxy)-germacran-8*α*,12-olide (**B**); cardivarolide H (**C**); 4*β*,8*α*-dihydroxy-5*β*-isobutyryloxy-9*β*-(3-methylbutyryloxy)-3-oxo-germacran-6*α*,12-olide (**D**); cardivarolide F (**E**); cardivarolide G (**F**) and 10-isobutyryloxy-8,9-epoxythymyl isobutyrate (**G**).

From the shoots grown on solidified MS medium supplemented with 0.5 µM TDZ five sesquiterpene lactones (see Figure 1), known constituents of *C. divaricatum*, were purified: 4*β*,8*α*-dihydroxy-5*β*,9*β*-diangeloyloxy-3-oxo-germacran-6*α*,12-olide (cardivarolide F), 4*β*,8*α*-dihydroxy-5*β*-isobutyryloxy-9*β*-angeloyloxy-3-oxo-germacran-6*α*,12-olide (cardivarolide H), 4*β*,8*α*-dihydroxy-5*β*-isobutyryloxy-9*β*-(3-methylbutyryloxy)-3-oxo-germacran-6*α*,12-olide, 2*α*,5-epoxy-5,10-dihydroxy-6*α*-isobutyryloxy-9*β*-(3-methylbutyryloxy)-germacran-8*α*,12-olide, and 2*α*,5-epoxy-5,10-dihydroxy-6*α*-angeloyloxy-9*β*-isobutyryloxy-germacran-8*α*,12-olide (isocardivarolide C). The compounds were identified based on their ^1^H NMR spectra in a reference to previously published data [5,8,19,20,21,22]. Moreover, ^1^H NMR spectrum of one from the isolated subfractions allowed for identification of 4*β*,8*α*-dihydroxy-5*β*-angeloyloxy-9*β*-(3-methylbutyryloxy)-3-oxo-germacran-6*α*,12-olide (cardivarolide G) [22] as another secondary metabolite produced by the shoots (Figure 1).

Chromatographic analysis of the extracts from in vitro cultured plants revealed the presence of 10-isobutyryloxy-8,9-epoxythymyl isobutyrate, the monoterpenoid formerly found in the root cultures of *Inula* spp. and multiple shoots of *Telekia speciosa* [16,17,23]. The compound (for the structure, see Figure 1) is a known metabolite of *C. divaricatum* [24].

**Figure 2:**
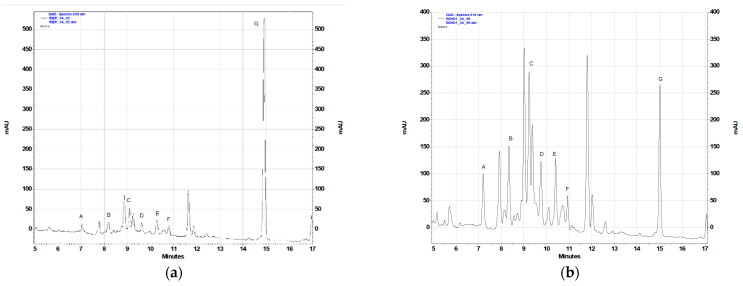

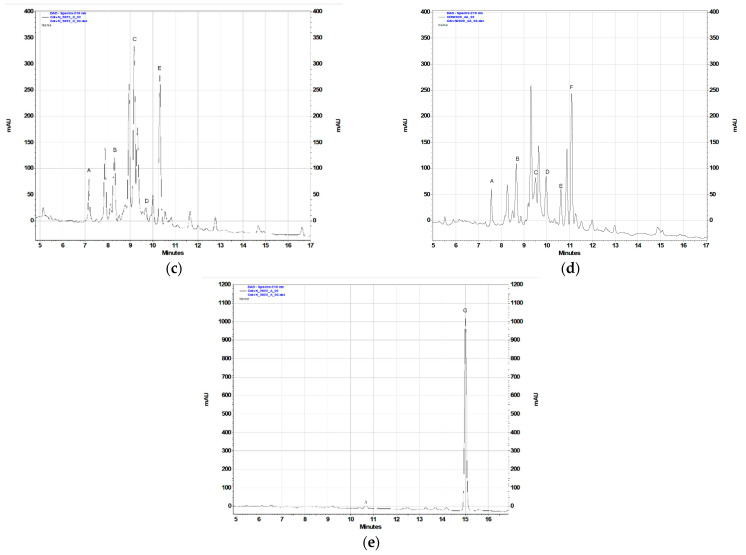
Chromatographic separation (RP-HPLC-DAD; λ = 205 nm) of *C. divaricatum* extracts (100 mg/mL): (**a**) plants from a liquid MS medium containing TDZ; (**b**) plants grown on a solidified MS medium supplemented with 2.0 μM BA and 0.1 μM NAA; (**c**) leaves of field-grown plants collected in 2022; (**d**) leaves of the plants collected in 2020; (**e**) roots of a field-grown plant collected in 2022.

To assess the contents of the identified terpenoid metabolites in the acetone extracts prepared from the plant material under study, the RP-HPLC-DAD method previously used for quantification of sesquiterpene lactones and 10-isobutyryloxy-8,9-epoxythymyl isobutyrate in *Telekia speciosa* [25] was applied (Table 2, Figure 2). The gradient profile was modified (see Section 4.4.2) to improve the resolution. The results of the analysis are shown in Table 2.

In the field-grown plants of *C. divaricatum*, germacranolides were major terpenoid constituents of aerial parts, whereas roots accumulated mainly 10-isobutyryloxy-8,9-epoxythymyl isobutyrate (Figure 2c,e). Extracts from the leaves collected in 2020 and in 2022 differed in their composition (Figure 2c,d). Extracts from the freshly collected leaves demonstrated higher contents of the compounds C and E, whereas extracts from the leaves collected in 2020 contained compound F (absent from the leaves collected in 2022). The cause of the difference may be disputable, but the effect of the storage period on the quality of the raw material is worth further studies. The extracts from roots of *C. divaricatum* collected in 2020 did not contain 10-isobutyryloxy-8,9-epoxythymyl isobutyrate (data not shown), probably due to the degradation of the compound.

*C. divaricatum* plantlets cultivated in vitro, in a liquid medium containing 0.5 µM TDZ (Figure 2a), accumulated lower amounts of germacranolides than the leaves of field-grown plants (Figure 2c,d) and the plantlets cultivated in vitro on solidified media supplemented with BA and NAA (Figure 2b), but contained a substantial amount of 10-isobutyryloxy-8,9-epoxythymyl isobutyrate. The plantlets grown on solidified media with addition of BA and NAA demonstrated a germacranolide accumulation pattern like that in leaves of the field-grown plants (Figure 2b–d).

Chromatographically analyzed extracts: 05TDZL, 20B01NS, CdN2020, CdN2022, and CdK2022 and the extracts used for cytotoxicity assays were prepared simultaneously, from the same batch of plant material and in the same manner.

### 2.3. Cytotoxic Activity of Extracts from In Vitro- and Field-Grown C. divaricatum

The assayed extracts, irrespectively of their chemical composition, exerted a cytotoxic effect on the cell lines used in the experiment (see Table 3). Moreover, the observed effect was dose- and time-dependent. The most susceptible to the action of extracts were Caco2 cells (IC_50_: 14.62–32.32 µg/mL, after 48 h) followed by HTB140 cells (IC_50_: 19.87–33.89 µg/mL, after 48 h). The most resistant were human keratinocytes (HaCaT; IC_50_ ≥ 75.64 µg/mL, after 48 h) and hepatoma cells (HepG2; IC_50_ ≥ 74.93 µg/mL, after 48 h). Some differences in the cytotoxic action of the examined extracts were observed. The extract from freshly collected leaves of plants grown in the open field was active against all cell lines used in the study (IC_50_: 27.43–98.41 µg/mL, after 24 h and IC_50_: 21.92–75.64 µg/mL, after 48 h) whereas the extract from leaves harvested in 2020 showed some selectivity of action on the cells. The difference may be connected with a distinct composition of the extracts (Figure 2c,d). Leaves of the field-grown plants contained no detectable amount of 10-isobutyryloxy-8,9-epoxythymyl isobutyrate. The compound was a major terpenoid constituent of freshly collected *C. divaricatum* roots. Despite the various composition (Figure 2c,e), both root and leaf extract were active in the assay, although their activities towards the individual cell lines differed. The extract from plantlets cultivated on a solidified nutrient medium, supplemented with BA and NAA, was especially active against PC3 and Caco2 cells (IC_50_ = 14.07 µg/mL and 14.62 µg/mL, respectively, after 48 h treatment). The extract contained both sesquiterpene lactones and the monoterpenoid thymol derivative (compound G).

## 3. Discussion

*C. divaricatum* is a species native to East Asia. Plants of the species cultivated in Central Europe due to late flowering fail to produce seeds. In vitro multiplication of shoots may provide plantlets for further cultivation in the open field or may be a good starting point for developing an in vitro culture system to produce biologically active plant metabolites. The multiple shoots cultivated in vitro may be also helpful in elucidation of the mechanisms implicated in the regulation of secondary metabolism of the plant.

Literature data on micropropagation of plants from the Inuleae tribe are sparse. The only study on in vitro culture of *Carpesium* [13] described changes in several physiological parameters of *C. triste* plantlets, induced by the color of light used to illuminate the cultures. The plantlets were grown on MS nutrient medium deprived of growth regulators and the data on shoot proliferation were not given. Nodal explants of *Inula royleana* DC., cultivated for 6 weeks on a solidified MS medium containing 5.0 μM kinetin and 0.1 μM NAA, regenerated 5.1 ± 1.9 shoots per explant [26]. Shoot tip explants of *Inula germanica* L., after 4 weeks of culture on MS medium supplemented with 4.44 μM BA and 0.54 μM NAA, produced from 3.4 ± 0.7 to 4.9 ± 0.9 shoots per explant. The number of regenerated shoots varied depending on the number of subcultures [27]. Similar multiplication rate (ca. 4 shoots/explant), after 4-week culture, was achieved for *T. speciosa*, a species closely related to *C. divaricatum*, using the same medium composition [16]. By extending the culture period to 8 weeks, the multiplication rate of *T. speciosa* had been increased over two times (up to 10.9 ± 3.1). The nutrient medium containing 4.44 μM BA and 0.54 μM NAA was less effective when applied to *C. divaricatum* shoot regeneration from nodal explants (multiplication rate after 8-week culture: 3.49 ± 1.22, see Table 1). Replacement of BA and NAA by TDZ (0.5 μM) did not increase the multiplication rate, but significantly increased the dry weight of plantlets regenerated from one explant (from 0.081 ± 0.017 to 0.131 ± 0.023). Liquid media supplemented with TDZ (0.5 or 1.0 μM) were more advantageous for both multiplication of shoots (although the effect was not statistically significant) and the dry weight increment. As the achieved multiplication rates were low, further optimization of the shoot multiplication procedure was needed.

In vitro cultivated shoots of the Asteraceae plants are usually a reliable source of those sesquiterpene lactones, which are accumulated by aerial parts of intact plants [28,29,30,31]. Multiple shoots and plantlets of *C. divaricatum* cultivated on solidified MS media containing BA and NAA and leaves of the field-grown plants accumulated comparable amounts of germacranolides (Figure 2, Table 2). Differences in terpenoid content observed in the in vitro cultivated plantlets (Table 2) may be caused by either different media composition or by the difference in the light regime. Biosynthesis of a pharmacologically active sesquiterpene lactone, artemisinin, by *Artemisia annua* L. tissue cultures was proven to be regulated by light [32,33]. In plant tissue cultures, fast growth is often accompanied with limited secondary metabolite production. The fast-growing *C. divaricatum* shoots, cultivated in liquid medium containing TDZ, produced less sesquiterpene lactones (as % DW) than the shoots grown on a solidified medium of the same composition.

Several dozen of sesquiterpene lactones were isolated from *C. divaricatum* plants, and germacranolides were the most frequently found metabolites of the group. Due to the co-occurrence of many structurally related compounds in the plant extract, development of the analytical method for quantification of *Carpesium* terpenoids in the raw extract is difficult. For a semi-quantitative estimation of terpenoid contents in the assayed plant extracts, the analytical method formerly used for quantification of sesquiterpene lactones and 10-isobutyryloxy-8,9-epoxythymyl isobutyrate in *T. speciosa* extracts [25] was adapted. The peak resolution was affected by the composition of the plant material under study and, to some extent, was improved by dilution of the sample.

The cell lines used for cytotoxicity evaluation were grouped in three panels, namely prostate, skin and gastrointestinal panels. Each panel comprised two cancer cell lines, differing in their metastatic potential, accompanied by non-neoplastic cell line of similar origin. The use of a non-neoplastic cells in the experiments gives the information on the selectivity of the tested samples. In the case of the gastrointestinal panel, hepatoma cell line (HepG2) was used instead of the appropriate non-neoplastic cells. This cell line is widely used in hepatotoxicity studies, as it reveals several phenotypic characteristics and many functional properties of normal liver cells [34,35].

The most susceptible cell line among all used in the study was colon adenocarcinoma Caco2, while the other colon adenocarcinoma cells (HT29) were more resistant. IC_50_ values estimated for the two cell lines were two- or threefold different. What is important, the HepG2 cells were almost unaffected, what indicates the selectivity of the extracts. As far as the prostate panel is concerned, the tested extracts were generally more active towards highly metastatic PC3 prostate carcinoma, compared to less metastatic DU145 prostate carcinoma. However, only low selectivity of the extracts was noted, as in most cases, non-neoplastic PNT2 prostate epithelial cells were also strongly affected. In the case of skin panel, the tested extracts were much more selective and revealed varied cytotoxic impact to two melanoma cell lines, while normal keratinocytes were affected to a lesser extent (IC_50_ > 75.6 µg/mL). Interestingly, highly metastatic HTB140 cells were more susceptible to the tested extracts compared to the other melanoma cell line (A375) used in the study that had less metastatic potential.

Sesquiterpene lactones are regarded as the biologically active metabolites, responsible for cytotoxic and anti-inflammatory activity of *Carpesium* plants [3]. Cytotoxic activity of the germacranolides isolated from *C. divaricatum* towards the human cancer cells in vitro was confirmed in several assays [3,8,12,21,22,36,37]. The compounds are accumulated in aerial parts of the plant and the plant roots seem to be almost deprived of them (Figure 2c–e). The plantlets cultivated in vitro, in MS liquid medium (containing 0.5 μM TDZ), were also poor producers of sesquiterpene lactones (Figure 2a). However, as it was shown in the present study, cytotoxic activity of the plant extracts and the sesquiterpene lactone content are not closely linked. Interestingly, traditional medicinal systems used whole herb of *C. divaricatum* as a remedy for various ailments [3]. The results of RP-HPLC-DAD analysis (Figure 2e) showed that 10-isobutyryloxy-8,9-epoxy-thymyl isobutyrate is a major terpenoid constituent of the plant roots. The compound has been recently described as an inhibitor of aberrant proliferative signaling in melanoma cells [38] and antileishmanial agent [39] and is known as a secondary metabolite synthesized by numerous species from the Asteraceae [40].

Acetone extracts from both in vitro- and field-grown plants of *C. divaricatum* are complex mixtures of active constituents that demonstrate diverse profiles of activity. Detailed knowledge of biochemistry of the plant, stability of the active metabolites and activity of their degradation products, new data on molecular mechanisms of action of isolated compounds combined with a development of a dedicated analytical method may lead to the better understanding of traditional use of *C. divaricatum* as a medicine and to potential future applications.

## 4. Materials and Methods

### 4.1. General Experimental Procedures

^1^NMR spectra were recorded in MeOD on a Bruker AVANCE III HD 400 (resonance frequency 400.17 MHz) spectrometer (Bruker Corp., Billerica, MA, USA). RP-HPLC separations were performed using an Agilent 1200 Series HPLC system (Agilent Technologies Inc., Santa Clara, CA, USA) equipped with a diode array detector. Analytical chromatographic separations were conducted on a Kinetex XB-C18 column (4.6 × 250 mm, 5 μm total particle size; Phenomenex Inc., Torrance, CA, USA). Semipreparative RP-HPLC was conducted on a Vertex Plus Eurospher II 100-5 C18 column (8 × 250 mm; Knauer GmbH, Berlin, Germany), with an isocratic elution, using methanol–water (MeOH-H_2_O) mixtures of different polarity. Conventional column chromatography (CC) was conducted on Silica gel 60 (0.063–0.2 mm, Merck KGaA, Darmstadt, Germany). Thin-layer chromatography (TLC) separations were performed using precoated plates (Silica gel 60 without fluorescence indicator, Art. No 5553, Merck, Darmstadt, Germany). Solvents of analytical grade were purchased from Avantor Performance Materials S.A. (Gliwice, Poland). Water was purified by a Mili-Q system (Milipore Corp., Bedford, MA, USA). MeOH and MeCN of HPLC grade were bought from Merck (Darmstadt, Germany).

### 4.2. Plant Material

#### 4.2.1. Field-Grown Plants

*C. divaricatum* plants were grown in the Garden of Medicinal Plants, Institute of Pharmacology, Polish Academy of Sciences, Kraków from seeds that were provided by the Research Center for Medicinal Plant Resources, National Institute of Biomedical Innovation (Tsukuba, Japan). Aerial parts of the plants were collected in the beginning of flowering period (August/September) and dried under shade at room temperature. A voucher specimen (3/15) was deposited in the collection kept at the Garden of Medicinal Plants, Institute of Pharmacology, Kraków, Poland.

#### 4.2.2. In Vitro Culture

Seeds of *C. divaricatum* were surface sterilized and aseptically germinated on a half-strength MS (Murashige and Skoog) medium [18], solidified with 0.8% agar. Nodal explants (ca. 0.08 g fresh weight, FW, each), excised from aseptic seedlings, were inoculated onto the three variants of solidified MS medium containing different combinations of plant growth regulators: (1) 0.5 μM TDZ (thidiazuron); (2) 2.0 μM BA (6-benzyladenine) and 0.1 μM NAA (1-naphtaleneacetic acid) or (3) 4.44 μM BA and 0.54 NAA. Alternatively, the nodal explants were inoculated in liquid MS medium supplemented with either 0.5 μM or 1 μM of TDZ. All nutrient media used in the experiments contained 3% sucrose and their pH was adjusted to 5.8, before autoclaving. The explants were kept at 28 °C, those on solidified media were maintained under continuous illumination (40 μmol m^−2^ s^−1^, cool white fluorescent tubes) and those in liquid media were cultivated under the 16/8 (light/dark) photoperiod (20 μmol m^−2^ s^−1^, cool white fluorescent tubes) with shaking (100 r.p.m.), to induce multiplication of shoots. After 12 weeks of culture, secondary explants (i.e., nodal explants excised from the regenerated shoots) were transferred to the fresh culture medium of the same composition as the induction medium, for further growth. Temperature and illumination conditions remained unaltered. The regenerated shoots were subcultured every eight weeks by inoculating nodal explants (ca. 0.1 g fresh weight) into the fresh nutrient medium. The biomass, containing regenerated shoots and plantlets, was collected at the end of the growth cycle and dried at room temperature prior to the phytochemical examination.

### 4.3. Extraction and Isolation of Sesquiterpene Lactones

Shoots and plantlets, collected from the culture on solidified MS medium containing 0.5 μM TDZ, were dried and powdered. The dry plant material (25.7 g) was extracted with CHCl_3_ (5 × 0.3 L). The organic extracts were combined and concentrated in vacuo to provide 1.07 g of an oily residue. The residue was fractionated by conventional column chromatography (CC) over silica gel (80 g) using an n-hexane–EtOAc gradient solvent system (up to 100% EtOAc). Collected fractions (50 mL each) were combined as shown by TLC.

Fractions 144–147 and 148–151, eluted with n-hexane–EtOAc 7:3 (*v*/*v*), were subjected to semipreparative RP-HPLC on a Vertex Plus Eurospher II 100-5 C18 column (eluent: MeOH–H_2_O mixture, 3:2, *v*/*v*, isocratic mode, flow rate: 2 mL/min) to yield cardivarolide F (23.8 mg; *t_R_*—68 min) and a mixture containing cardivarolide G (20.9 mg; *t_R_*—85 min). Fractions 148–151 gave additionally cardivarolide H (3 mg; *t_R_*—54 min).

Fractions 152–154 (eluted with n-hexane-EtOAc; 7:3, *v*/*v*), after purification by semipreparative RP-HPLC (as described above), provided 4*β*,8*α*-dihydroxy-5*β*-isobutyryloxy-9*β*-(3-methylbutyryloxy)-3-oxo-germacran-6*α*,12-olide (1.2 mg, *t_R_*—28 min). Fractions 166–172 and 173–179, eluted with n-hexane-EtOAc 3:2 (*v*/*v*), were submitted to chromatographic separation by RP-HPLC (eluent: MeOH-H_2_O mixture, 3:2, *v*/*v*, isocratic mode, flow rate: 1.5 mL/min) and yielded 2*α*,5-epoxy-5,10-dihydroxy-6*α*-isobutyryloxy-9*β*-(3-methylbutyryloxy)-germacran-8*α*,12-olide (1 mg; *t_R_*—53 min) and isocardivarolide C (3.2 mg; *t_R_*—40 min), respectively.

Isolated compounds were identified based on their ^1^H NMR spectra and comparison of the experimentally obtained data with those found in the literature [5,8,19,20,21,22].

### 4.4. RP-HPLC-DAD Analysis of Terpenoids in Plant Extracts

#### 4.4.1. Sample Preparation

The dry and pulverized plant material (0.1 g) was extracted twice with 10 mL of acetone at room temperature for 3 h on a rotary shaker (100 r.p.m.). The extracts from the two consecutive extractions were combined and evaporated to dryness under reduced pressure to give a residue that was redissolved in 1 mL of MeOH and centrifuged (11,340× *g*, 5 min) prior to HPLC analysis.

#### 4.4.2. Semi-Quantitative Assessment of 10-Isobutyryloxy-8,9-epoxythymyl Isobutyrate and Sesquiterpene Lactone Content

Isocardivarolide C (A); 2*α*,5-epoxy-5,10-dihydroxy-6*α*-isobutyryloxy-9*β*-(3-methylbutyryloxy)-germacran-8*α*,12-olide (B); cardivarolide H (C); 4*β*,8*α*-dihydroxy-5*β*-isobutyryloxy-9*β*-(3-methylbutyryloxy)-3-oxo-germacran-6*α*,12-olide (D); cardivarolide F (E); cardivarolide G (F) and 10-isobutyryloxy-8,9-epoxythymyl isobutyrate (G) were identified in the examined plant extracts based on their retention times, UV spectra and co-chromatography with the formerly isolated *C. divaricatum* germacranolides [E1] and the thymol derivative isolated from *T. speciosa*. Their content in the investigated plant material was estimated using the analytical method described previously [25] with 2,3-dihydroaromaticin and 10-isobutyryloxy-8.9-epoxythymyl isobutyrate as external standards. Peak areas were measured at 205 nm. To improve resolution of the signals, the gradient timetable was slightly modified, and some samples had to be diluted (2× or 4×). Components of the mobile phase: water (A) and MeCN (B) remained unaltered but the linear gradient from 40% B to 75% B in 20 min, and 98% B in another 5 min was applied (stop time: 35 min, post time: 15 min). The remaining parameters of the method remained unchanged.

### 4.5. Cell Culture and Cytotoxicity Assessment

#### 4.5.1. Preparation of Plant Extracts

The dry and pulverized plant material (1 g) was extracted two times (3 h) with 100 mL of acetone, at room temperature, with shaking. The combined extracts were concentrated in vacuo to remove the solvent. From the dry residues, the stock solutions in MeOH (10 mg/mL) were prepared. The working concentrations of the extracts (from 5 to 100 μg/mL) were obtained by dilution of the stock solutions with the culture media.

#### 4.5.2. Assessment of Cytotoxic Activity

The experiment was performed on several human cancer and normal cell lines, grouped as follows: skin panel (melanoma HTB140, derived from metastatic site: lymph node, ATCC Hs 294T; malignant melanoma A375, ATCC CRL-1619; skin keratinocytes HaCaT), prostate panel (prostate carcinoma DU145, derived from metastatic site: brain, ATCC HTB-81; grade IV prostate carcinoma PC3, derived from metastatic site: bone, ATCC CRL- 1435; prostate epithelial cells PNT2, ECACC 95012613), and gastrointestinal panel (colorectal adenocarcinomas Caco-2, ATCC HTB-37 and HT-29, ATCC HTB- 38; hepatocellular carcinoma HepG2, ATCC HB-8065). The cells were cultured in humidified atmosphere in CO_2_ incubator, at 37 °C, on MEM low glucose (DU145), MEM high glucose (HTB140, A375, HaCaT), DMEM/F12 (HT29, PC3, PNT2, HepG2), and MEM with NEAA (Caco2), with 10% of bovine serum and antibiotics, as described previously [41]. Cells were seeded in 96-well plates for 24 h (1.5 × 10^4^ cells/well), and then a fresh medium containing different concentrations of the tested extracts (5–100 µg/mL) was added. The incubation lasted 24 and 48 h. Cell viability was measured by MTT assay, as previously described [42]. The absorbance at 490 nm was measured using Synergy II Biotek (BioTek Instruments, Winooski, VT, USA) microplate reader. Cytotoxic activity was assessed based on cell viability expressed as percent of living cells. Results were means of three independent measurements (±SD). Doxorubicin (Ebewe Pharma GmbH., Unterach, Austria) was used as a reference cytostatic drug. The IC_50_ values were determined by plotting the percentage viability of the cells versus concentration and the adequate calculations made using either Excel or AAT Bioquest website program (https://www.aatbio.com/tools/ic50-calculator accessed on 4 September 2022).

### 4.6. Statistical Analysis

Data were analyzed using the Statistica software (v.13.3). The analysis of variance (one-way ANOVA) and post hoc Tukey test were used to show statistical significance with assumed *p* < 0.05.

## 5. Conclusions

In vitro cultivated shoots and plantlets of *C. divaricatum* produced terpenoids characteristic of the intact plant and the extracts from the in vitro cultured plant material demonstrated cytotoxic activity comparable to that of extracts from the field-grown plant. Taking into consideration the results of RP-HPLC-DAD analysis of the assayed extracts and results of the cytotoxicity assessment, the activity of extracts from both in vitro- and field-grown plants can not be unambiguously assigned to the presence of sesquiterpene lactones. Extracts of different origin demonstrated various specificity towards the cancer cells used in the study. The complicated correlation of the extract composition and cytotoxic activity should be a subject of further research.

## Figures and Tables

**Table 1:** Effect of the nutrient medium and light conditions on in vitro multiplication of shoots from the nodal explants of *Carpesium divaricatum* after 8 weeks of culture.

Culture Medium	Light Conditions	Regeneration of Shoots (%)	Number of Shoots per Explant	Final Dry Weight per Explant (g) ^3^
MS + 0.5 μM TDZ; solidified medium ^1^	continuous illumination; cool white light; 40 μmol m^−2^ s^−1^	100	3.36 ± 1.50 ^1,a^	0.131 ± 0.023 ^b^
MS + 2.0 μM BA + 0.1 μM NAA; solidified medium ^1^	continuous illumination; cool white light; 40 μmol m^−2^ s^−1^	96.8	3.73 ± 1.43 ^1,a^	0.067 ± 0.014 ^a^
MS + 4.44 μM BA + 0.54 μM NAA; solidified medium ^1^	continuous illumination; cool white light; 40 μmol m^−2^ s^−1^	94.5	3.49 ± 1.22 ^1,a^	0.081 ± 0.017 ^a^
MS + 0.5 μM TDZ; liquid medium ^2^	photoperiod 16/8 (light/dark); cool white light; 20 μmol m^−2^ s^−1^	100	3.78 ± 1.37 ^2,a^	0.216 ± 0.042 ^c^
MS + 1.0 μM TDZ; liquid medium ^2^	photoperiod 16/8 (light/dark); cool white light; 20 μmol m^−2^ s^−1^	100	4.07 ± 1.61 ^2,a^	0.269 ± 0.028 ^c^

^1^ Data from seven consecutive passages (n = 61), mean ± SD; ^2^ Data from five consecutive passages (n = 27), mean ± SD; ^3^ Mean of four independent measurements ± SD. Results denoted with the same letter were not statistically different (*p* > 0.05; one-way ANOVA).

**Table 2:** Contents of the identified sesquiterpene lactones (A–F) and thymol derivative (G) semi-quantitatively estimated in the plant material from in vitro culture and in the organs of field-grown plants.

Plant Extract	Contents of the Terpenoids (% DW) ^1^						
	A	B	C	D	E	F	G
05TDZL ^2^	0.016 ± 0.002 ^a^	0.026 ± 0.002 ^a^	0.036 ± 0.004 ^a^	0.021 ± 0.003 ^a^	0.027 ± 0.004 ^a^	0.018 ± 0.003 ^a^	0.064 ± 0.008 ^a^
05TDZS	0.034 ± 0.003 ^a,b^	0.049 ± 0.006 ^b^	0.018 ± 0.001 ^b^	0.083 ± 0.004 ^b^	0.040 ± 0.005 ^a^	0.094 ± 0.016 ^b^	0.040 ± 0.001 ^b^
20B01NS	0.051 ± 0.004 ^b,c^	0.114 ± 0.002 ^c^	0.138 ± 0.001 ^c^	0.081 ± 0.002 ^b^	0.104 ± 0.002 ^b^	0.034 ± 0.001 ^a^	0.043 ± 0.004 ^b^
44B05NS	0.077 ± 0.010 ^d^	0.039 ± 0.001 ^b^	0.023 ± 0.001 ^b^	0.139 ± 0.019 ^c^	0.079 ± 0.002 ^c^	0.214 ± 0.021 ^c^	0.028 ± 0.004 ^b^
CdN2020	0.047 ± 0.001 ^b^	0.079 ± 0.006 ^d^	0.031 ± 0.001 ^a^	0.070 ± 0.002 ^b^	0.049 ± 0.001 ^a^	0.179 ± 0.007 ^c^	nd
CdN2022	0.060 ± 0.007 ^c,d^	0.089 ± 0.006 ^d^	0.171 ± 0.004 ^d^	0.018 ± 0.001 ^a^	0.201 ± 0.013 ^d^	nd	nd
CdK2022	nd	nd	nd	nd	nd	nd	0.118 ± 0.008 ^c^

^1^ Values are means of three measurements (±SD); the means denoted by the same letter did not differ significantly (*p* > 0.05). Statistical analysis was done with one-way ANOVA with Tukey’s post hoc test; nd—not detected. ^2^ 05TDZL—liquid MS medium containing 0.5 μM TDZ; 05TDZS—solidified MS medium containing 0.5 μM TDZ; 20B01NS—solidified MS medium containing 2.0 μM BA and 0.1 μM NAA; 44B05NS—solidified MS medium containing 4.44 μM BA and 0.54 μM NAA; CdN2020—leaves of the field-grown plants collected in 2020; CdN2022—leaves of the field-grown plants collected in 2022; CdK2022—roots of the field-grown plants collected in 2022.

**Table 3:** Cytotoxic activities of extracts from in vitro- and field-grown *Carpesium divaricatum*, after 24 and 48 h. Doxorubicin was used as a reference cytostatic; 05TDZL—plants cultivated in a liquid MS medium containing 0.5 μM TDZ; 20B01NS—plants cultivated on a solidified MS medium containing 2.0 μM BA and 0.1 μM NAA; CdN2020—leaves of the field-grown plants collected in 2020; CdN2022—leaves of the field-grown plants collected in 2022; CdK2022—roots of the field-grown plants collected in 2022.

Plant Extract and Time of Exposure (h)	IC_50_ (μg/mL) ^1^								
	Prostate Panel			Skin Panel			Gastrointestinal Panel		
	PNT2	Du145	PC3	HaCaT	A375	HTB140	HT29	Caco2	HepG2
05TDZL 24	27.48 ± 1.12 ^a^	79.18 ± 2.96 ^b^	44.94 ± 1.08 ^c^	>100	34.96 ± 1.02 ^a^	37.94 ± 1.24 ^b^	46.53 ± 1.51 ^a^	28.51 ± 0.98 ^b^	>100
05TDZL 48	21.42 ± 1.24 ^a^	54.59 ± 2.06 ^b^	32.63 ± 1.41 ^c^	>100	30.40 ± 0.80 ^a^	33.89 ± 1.06 ^b^	42.11 ± 1.03 ^a^	25.64 ± 0.74 ^b^	>100
20B01NS 24	43.17 ± 1.77 ^a^	76.24 ± 2.79 ^b^	26.50 ± 0.77 ^c^	>100	42.29 ± 1.33 ^a^	33.97 ± 1.41 ^b^	64.78 ± 1.81 ^a^	26.13 ± 1.04 ^b^	>100
20B01NS 48	30.23 ± 1.79 ^a^	42.48 ± 1.33 ^b^	14.07 ± 0.76 ^c^	94.15 ± 3.38 ^a^	32.07 ± 1.11 ^b^	19.87 ± 0.72 ^c^	52.76 ± 1.92 ^a^	14.62 ± 1.41 ^b^	>100
CdN2020 24	47.04 ± 1.84 ^a^	45.59 ± 1.77 ^a^	29.00 ± 1.41 ^b^	>100	68.04 ± 2.59 ^a^	39.06 ± 1.12 ^b^	47.56 ± 1.00 ^a^	24.64 ± 1.57 ^b^	>100
CdN2020 48	32.89 ± 1.30 ^a^	24.19 ± 0.95 ^b^	21.18 ± 1.33 ^b^	>100	42.91 ± 1.50 ^a^	26.12 ± 1.20 ^b^	21.18 ± 1.33 ^a^	16.21 ± 1.42 ^b^	98.27 ± 2.95
CdN2022 24	34.88 ± 1.10 ^a^	74.36 ± 1.91 ^b^	64.48 ± 1.59 ^c^	95.05 ± 3.85 ^a^	49.91 ± 1.58 ^b^	27.43 ± 1.09 ^c^	58.45 ± 1.87 ^a^	37.42 ± 2.29 ^b^	98.41 ± 3.49 ^c^
CdN2022 48	29.69 ± 1.28 ^a^	53.56 ± 1.54 ^b^	54.04 ± 1.97 ^b^	75.64 ± 3.06 ^a^	39.79 ± 2.39 ^b^	21.92 ± 1.04 ^c^	49.06 ± 1.79 ^a^	26.36 ± 2.04 ^b^	74.93 ± 2.12 ^c^
CdK2022 24	50.62 ± 1.86 ^a^	80.61 ± 3.34 ^b^	43.61 ± 1.98 ^c^	>100	74.14 ± 2.27 ^a^	46.13 ± 1.29 ^b^	75.00 ± 2.52 ^a^	53.93 ± 1.97 ^b^	>100
CdK2022 48	33.81 ± 1.47 ^a^	61.28 ± 1.58 ^b^	34.56 ± 2.28 ^a^	>100	68.02 ± 2.32 ^a^	28.17 ± 1.80 ^b^	64.84 ± 1.64 ^a^	32.32 ± 1.47 ^b^	>100
Doxorubicin 24	1.38	3.18	>50	4.68	0.59	5.71	1.53	3.44	1.03

^1^ Mean of three independent measurements ± SD. Results denoted by the same letter were not statistically different (*p* > 0.05; one-way ANOVA). Comparisons were made separately for each extract, treatment duration, and each panel of the cells.

## Data Availability

The raw data from the study will be made available by the authors on request from any qualified researcher.
